# Electroless Ionization Mass Spectrometry Using a Compact Electrokinetic Ionization Source

**DOI:** 10.1021/acs.analchem.4c01403

**Published:** 2024-06-25

**Authors:** Stefan Kooij, Aleksandra Chojnacka, Daniel Bonn, Garry L. Corthals, Cees J. M. van Rijn

**Affiliations:** † Van der Waals-Zeeman Institute, University of Amsterdam, Science Park 904, 1098 XH Amsterdam, The Netherlands; ‡ Van ’t Hoff Institute for Molecular Sciences, University of Amsterdam, Science Park 904, 1098 XH Amsterdam, The Netherlands

## Abstract

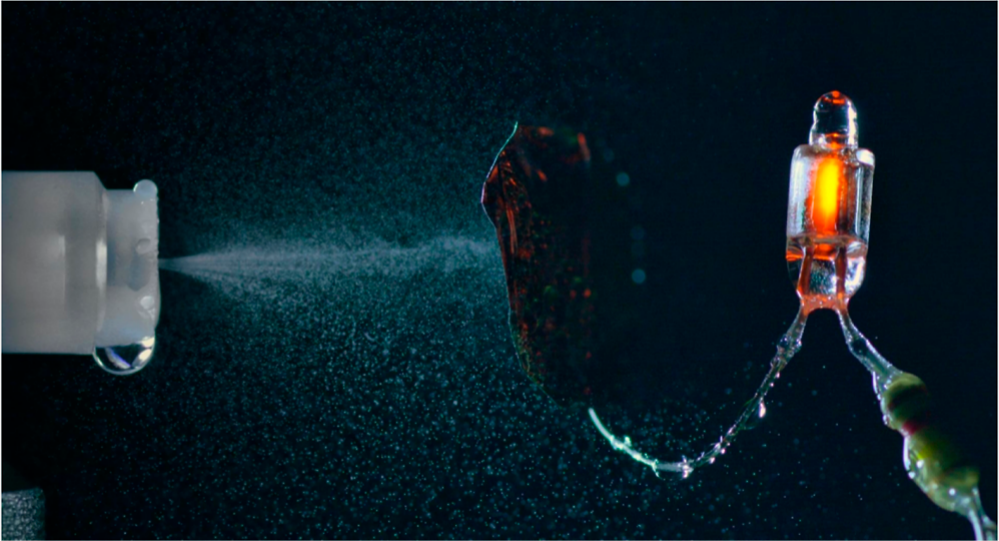

We introduce a new ionization technique for compact, portable mass spectrometers. It consists of a syringe with sample liquid capped by a self-ionizing spray nozzle containing a microfabricated nozzle chip. Interaction of the sample liquid with the nozzle wall results in electrical charging without the need for electronics. Elaborate cleaning procedures are redundant when disposable syringes and mass-fabricated spray nozzles are used. This self-named electroless spray ionization (ELI) technique shows comparable performance to conventional ionization techniques. In contrast to commonly used electrospray ionization, ELI exhibits excellent ionization efficiency for low-conductive solutions such as water or acetonitrile. Due to its compact size and the absence of high-voltage electronics, it can also be readily integrated in other ionization sources. Besides reviewing the main properties of ELI, we showcase the technique’s potential for two on-site, ambient mass spectroscopy applications: perfume fingerprinting and fast screening of fungicides on citrus fruits.

## Introduction

Since the early 90s, mass spectrometry (MS) has found its way into many applications and domains,^1−6^ owing to both the exceptional sensitivity of the instruments and the continual stream of innovations in MS. The introduction of ambient MS, where the sample is ionized under atmospheric conditions, has led to the development of direct sampling techniques that do not require extensive sample preparation. New ambient MS techniques have been developed,^7−11^ with most innovations covering the ionization step. Today’s ionization methods predominantly rely, in one way or another, on a (high) voltage supply.

**Figure 1:**
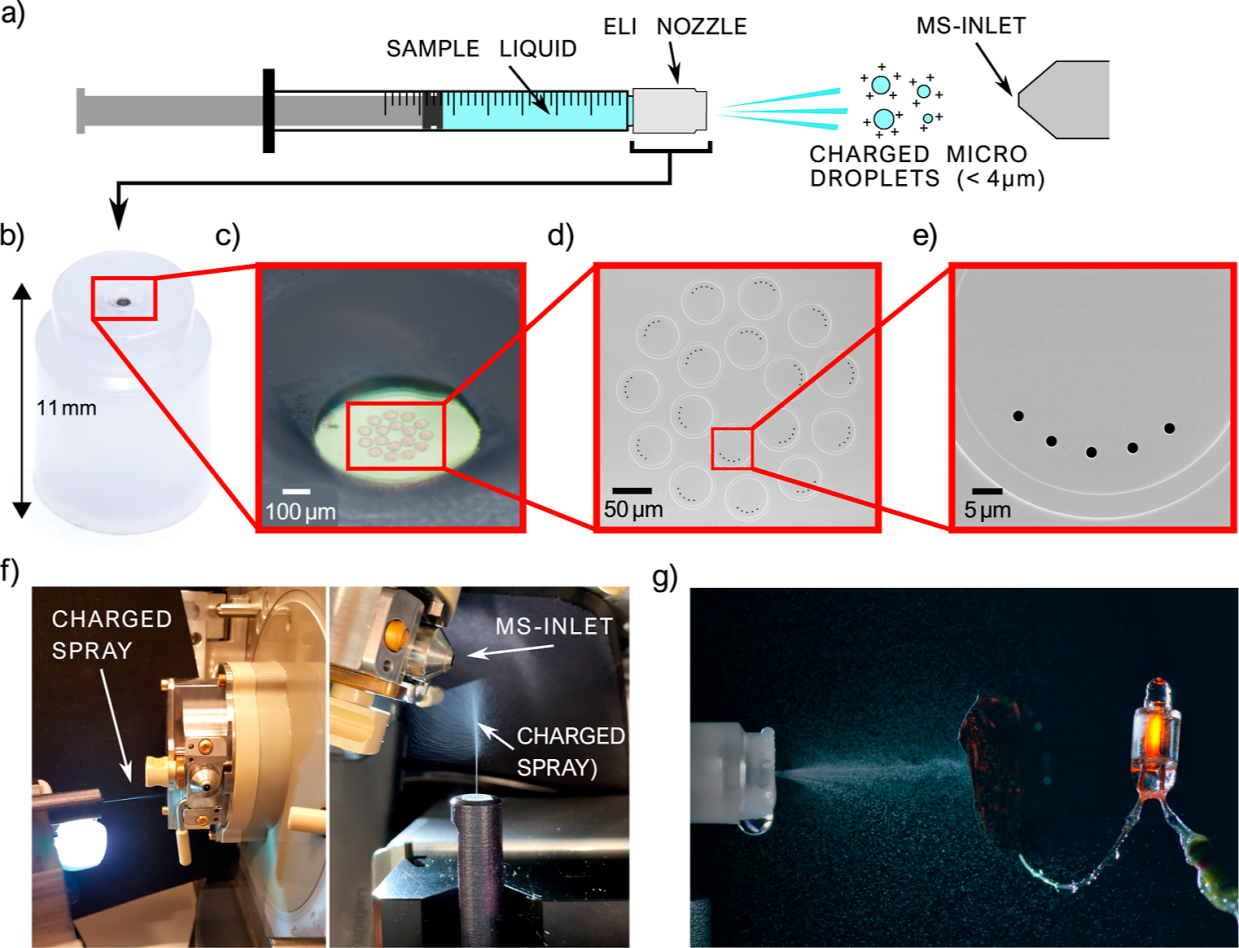
ELI-MS method. (a) Spray nozzle is fitted on top of a 1 mL syringe containing the sample of interest. By applying sufficient pressure, either manually or with a syringe pump, a cloud of charged micro droplet is formed. The charged spray is then directed toward the MS inlet, several cm from the ELI nozzle. (b–e) ELI nozzle comprises a polypropylene adapter of height 11 mm containing a 1 mm silicon chip, with 85 holes of 1.9 μm diameter. The pictured nozzle chip (c) contains 17 circular windows of 50 μm diameter (d), each containing 5 1.9 μm diameter holes in a thin layer of silicon nitride. (f) Picture of the ELI-nozzle with the MS-inlet. (g) ELI nozzle powering a small neon glow light. The ELI-charged droplets impact a metallic plate connected to ground via the neon lamp.

In this work, we introduce a novel ionization technique that does not require any electronics to produce and ionize droplets of sample liquid. This so-called electroless ionization (ELI) technique allows full control of drop size, charge polarity, and flow rates. In ELI, the sample liquid is pushed through a microfabricated spray nozzle (Figure 1). Due to the electrokinetic interaction with the nozzle surface, the liquid becomes charged.^12^ Combined with rapid evaporation, this charging results in a quickly expanding cloud of ionized particles for subsequent MS analysis.

The abundance of different ionization techniques in the field of MS demonstrates that in many processes, spontaneous charge separation occurs. Some interesting examples of other ionization techniques that work without the direct use of a high voltage supply are thermospray,^13^ zero volt paper spray,^14^ and inlet ionization techniques such as SAII^15−17^ and DAII.^18,19^ For thermospray and inlet ionization, the ionization mechanism is thought to result from a combination of statistical droplet charging, ion evaporation, and chemical ionization processes.^15,17,20^ The formation of molecular ions from charged droplets encompasses various elements such as, droplet evaporation, Coulomb fission, molecular ion ejection, and matrix effects. Though this process is still not fully understood, the biggest uncertainty for these techniques in the ion formation is the droplet charge and size distribution. For ELI, however, the droplet size and expected level of charging can be accurately predicted if the zeta potential between the nozzle and liquid is known.^12^ Optimization of the charging mechanism by making use of different nozzle materials is still an active area of research.

As we show here, ELI has specific advantages over conventional ionization methods. For example, it is very compact, ionization is efficient even when the conductivity of the solvent (mixture) is low, and the flow rates and ion currents are easily adjusted. ELI could therefore be used as a stand-alone, hand-held disposable ionization source but also be implemented in MS applications. The simplicity and compactness and the fact that no elaborate cleaning procedures are needed make the ELI technique ideal for fast measurements and nonexpert use, for example, in on-site and fieldable MS applications such as forensic investigations, airport security, and food quality control. Still, in many instances, such as when highly conductive liquids are used, techniques such as ESI are likely to produce superior results due to their higher level of ionization. Integration of the ELI nozzle chip in other applications, such as LC–MS, is an interesting direction that requires further research and development.

Here, we compare ELI to existing ionization techniques and show that the method can be used for quantitative MS analysis. We further show that by altering the surface charge of the interior of the nozzle, the polarity of the droplet charge can be reversed, an essential step in making this method broadly applicable. Finally, as the charging depends on the interaction of the solvent with the nozzle interior by a previously described mechanism,^12^ we provide more details on the parameters that play a role in the charging efficiency, such as fluid type, injection rate, and salt concentration.

## Experimental Section

### Setup

The ELI-MS setup is depicted in Figure 1. A nozzle (ion sprays) is fitted onto a 1 mL syringe (BD Plastipak). It should be noted that many disposable syringe brands contain contaminants, especially the lubricant oleamide. The BD Plastipak syringes were the only ones we tested that did not contain these contaminants. For reproducibility, we use a syringe pump, although operating the syringe manually is also possible. The spray plume is directed toward the MS inlet at a distance of a few cm and slightly to the side as not to spray directly into the MS inlet. Since ELI-MS does not require an external voltage supply and therefore does not contain a polarity switch, we adjusted the nozzle design and choose solvents or solvent mixtures to allow for either positive or negative ionization. The charging polarity and efficiency is mostly determined by the zeta potential between the nozzle’s charge transfer layer and the solvent or solvent mixture.^12^ For most common solvents or solvent mixtures such as water, acetonitrile, dimethyl sulfoxide, H_2_O/EtOH (v/v % water > 25), and H_2_O/MeOH (v/v % water > 25), the zeta potential between the liquid and the “positive” ELI nozzle is negative, resulting in positive ionization. For the “negative” ELI nozzle, the zeta potential is positive.

### MS Experiments

ELI-MS experiments were performed using a Synapt G2 MS (Waters, Milford, United States) or a Q Exactive MS (Orbitrap) under ambient native conditions without a commercial source attached. For the Synapt G2 MS, the analysis was carried out using an interface temperature of 120 °C, a desolvation gas flow of 5 L h^–1^, and a mass spectral range of *m*/*z* = 100–500 or 300–1200. The spectra were acquired using an accumulation time of 15 s, for full spectrum at the resolution 10,000 at *m*/*z* = 556. To compare ELI-MS with conventional methods, we performed electrospray ionization (ESI) experiments, where the capillary voltage was set to 3 kV, the desolvation temperature to 350 °C, and the cone gas flow to 800 L h^–1^. For the Q Exactive (Orbitrap), the sheath gas flow rate was 12 for ESI and 0 for ELI, spray voltage either 3.5(+) kV or 2.5(−) kV, the capillary temperature 320 °C (ESI) or 250 °C (ELI), the S-lens RF level 50, and the auxiliary gas flow 0. Heated ESI (HESI) experiments were performed at an injection flow rate of 10 μL min^–1^.

### Materials

To evaluate the performance of ELI-MS, acetaminophen (paracetamol), budesonide, caffeine, melittin, peptide mixture (GLY-TYR, VAL-TYR-VAL, methionine enkephalin, leucine enkephalin, and angiotensin II), and leucine enkephalin standards were purchased from Sigma-Aldrich (Burlington, MA, USA). ULC–MS-grade acetonitrile (ACN) and water (H_2_O), as well as HPLC-grade ethanol (EtOH) and isopropanol (IPA) from Biosolve (Valkenswaard, The Netherlands), were used to dissolve the standards. All solvents have been prepared in volume %.

### Grounding

Charging requires a closed electric circuit as a result of charged droplets leaving the nozzle, the nozzle itself becomes oppositely charged, attracting the spray. Though the syringe and nozzle are not grounded, the whole setup can still attract/repel sufficient electrons from the surroundings to maintain a constant level of charging during the measurement time on the order of minutes. By grounding the nozzles, which is achieved by applying a gold layer on the nozzle that is attached to electrical ground, the charging efficiency and stability of the spray can be increased. However, for simplicity, we used nongrounded ELI nozzles.

### Clogging

The ELI nozzles contain two built-in filters before the nozzle chip to prevent it from clogging. We verified that standard LC–MS sample preparation procedures result in liquids that do not clog these filters, allowing hours of continuous spraying.

## Results and Discussion

### ELI vs ESI

To compare the spectra for ESI and ELI, we used leucine enkephalin (555.62 g mol^−1^), which is a commonly used peptide for MS tuning and calibration. 20 ng mL^–1^ of leucine enkephalin dissolved in a 50/50 solution of ACN/H_2_O was characterized by both methods.

**Figure 2:**
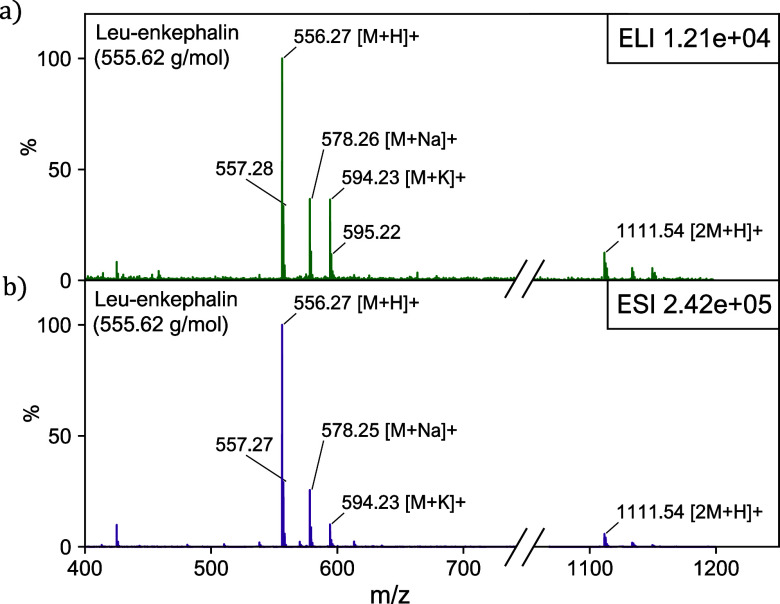
Mass spectra of a solution of 20 ng mL^–1^ leucine enkephalin in ACN/H_2_O (50/50), obtained from ELI-MS (a) and ESI-MS (b). Molecular ion is the most abundant in both cases, followed by the sodium and potassium adducts. The two spectra are equivalent, with a higher ion count for ESI-MS.

Similar spectra are obtained, as shown in Figure 2. Note that a comparison of experimental parameters such as the measurement time and flow rate is not meaningful, given that one method uses an open source and the other a closed source. The overall ion count for ESI-MS is an order of magnitude higher than for ELI-MS, reflecting the fact that the former is a closed source using an external high-voltage supply.

### Charging Efficiency

For the different solvents used in the experiments, we find that both the “positive” and “negative” ELI nozzles charge well, with acetonitrile giving the best ionization. Quantification of such differences would require a customized source for the ELI nozzles, which falls outside of the scope of the present study. Other charge transfer layers could provide much larger zeta potentials and therefore better charging efficiencies. The charging efficiency of ELI is best evaluated by a direct comparison with the widely used ESI. For a true comparison, however, an ELI nozzle optimized for maximum charging and low flow rates should be used as a closed ionization source, as is done in ESI. This is beyond the scope of this work, where we only use separate ELI nozzles suitable for ambient MS. As it is well understood what determines the level of charging for ELI,^12^ theoretical considerations do, however, allow one to estimate how an ELI-type nozzle would perform under such circumstances. In electrospray, using a flow rate of 5 μL min^–1^, the spray current can be 20 nA for solutions with a sample concentration on the order of 1 μM in a clean solvent.^21^ While higher spray currents can be obtained if solutions with more electrolytes are used, such concentrated solutions are not suitable for ELI-MS. The expected induced electric current *I*_s_ due to electrokinetic charging by a single ELI nozzle is given by^12^
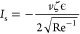
1where *v* is the liquid velocity, ζ the zeta potential between the nozzle charge transfer layer and the liquid, ϵ the electric permittivity of the liquid, and Re the Reynolds number given by Re = ρ*vR*/μ, where *R* is the nozzle hole radius, ρ the liquid density, and μ the viscosity of the liquid. For water, a single nozzle hole radius of 0.95 μm and a flow rate of 5 μL min^–1^, *I*_s_ = 6 nA, a value very similar to ESI. For higher ζ, *I*_s_ will further increase. To put these numbers into perspective, Figure 1g depicts a ‘positive’ ELI nozzle powering a small neon lamp, demonstrating that the electroless ionization is a significant effect.

In the above calculation, it is assumed that the ELI nozzle is grounded. We, however, make use of nozzle chips that are mounted inside a plastic adapter, such that one can expect that the electrical grounding is far from optimal. Nevertheless we find that a constant level of charging can be obtained with an intensity that is more than enough to ionize and disperse a sample. The setup as a whole provides sufficient electrical conductance to maintain charging during the measurement time of several minutes. To explore the effect of grounding, we applied a thin layer of gold on the exterior and interior of an ELI nozzle adapter and connected the adapter to electrical ground. We find that the overall intensity is increased, and the ion current shows a more stable profile (see Supporting Information Charging Stability and Grounding).

### Charging Polarity

**Figure 3:**
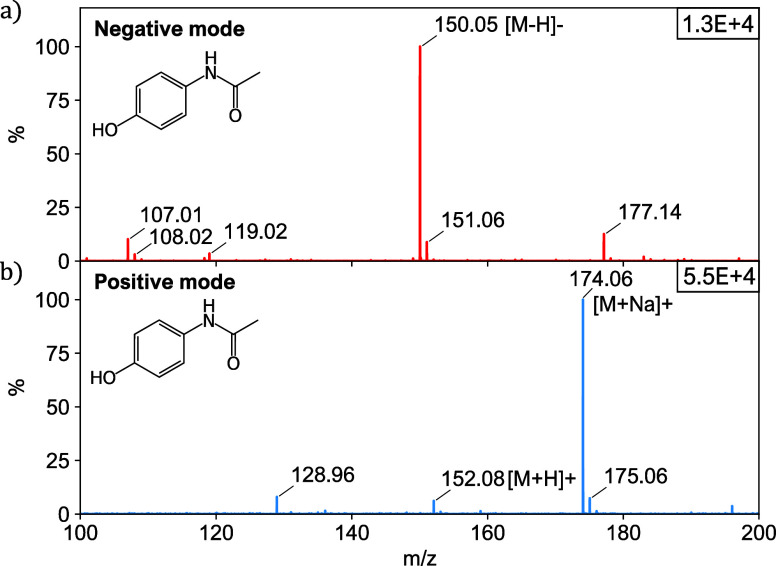
Mass spectra of paracetamol (151.163 g mol^−1^) in a 50/50 H_2_O/EtOH mixture using ELI-MS in negative (a) and positive (b) mode. In negative mode, the molecular ion, [M – H]^−^, is clearly visible. In positive mode, the main peak is the sodium adduct [M + Na]^+^.

To demonstrate that ELI-MS can be used for both positive as well as negative ionization, we use a sample solution of paracetamol (151.163 g mol^−1^), dissolved in a 50/50 mixture of H_2_O/EtOH. Figure 3 shows the mass spectrum of the sample in both modes. The polarity of the charging is set by the charge transfer layer in the nozzle, which determines the zeta potential between this layer and the solvent(s) used. The molecular ion [M – H]^−^ is clearly visible in negative mode, while no other compounds are found in the spectrum (100–700 Da). Positive-mode ELI-MS was also tested with ACN/H_2_O and IPA/H_2_O, yielding similar results (not shown).

### Charging Stability

To investigate the charging stability of ELI-MS, we measured the charging efficiency of an ELI nozzle with a single pore in positive mode by spraying demineralized water for 24 h. We found no change in the level of charging.

### ELI vs HESI

To compare ELI with HESI, we used a Q Exactive MS (Orbitrap) to measure a peptide mixture consisting of A/GLY-TYR (238.25 g mol^−1^), B/VAL-TYR-VAL (379.51 g mol^−1^), C/methionine enkephalin (573.68 g mol^−1^), D/leucine enkephalin (555.63 g mol^−1^), and E/angiotensin II (1046.19 g mol^−1^) in a 50/50 mixture of MeOH/H_2_O at a concentration of 5 μg mL^–1^ for each component.

**Figure 4:**
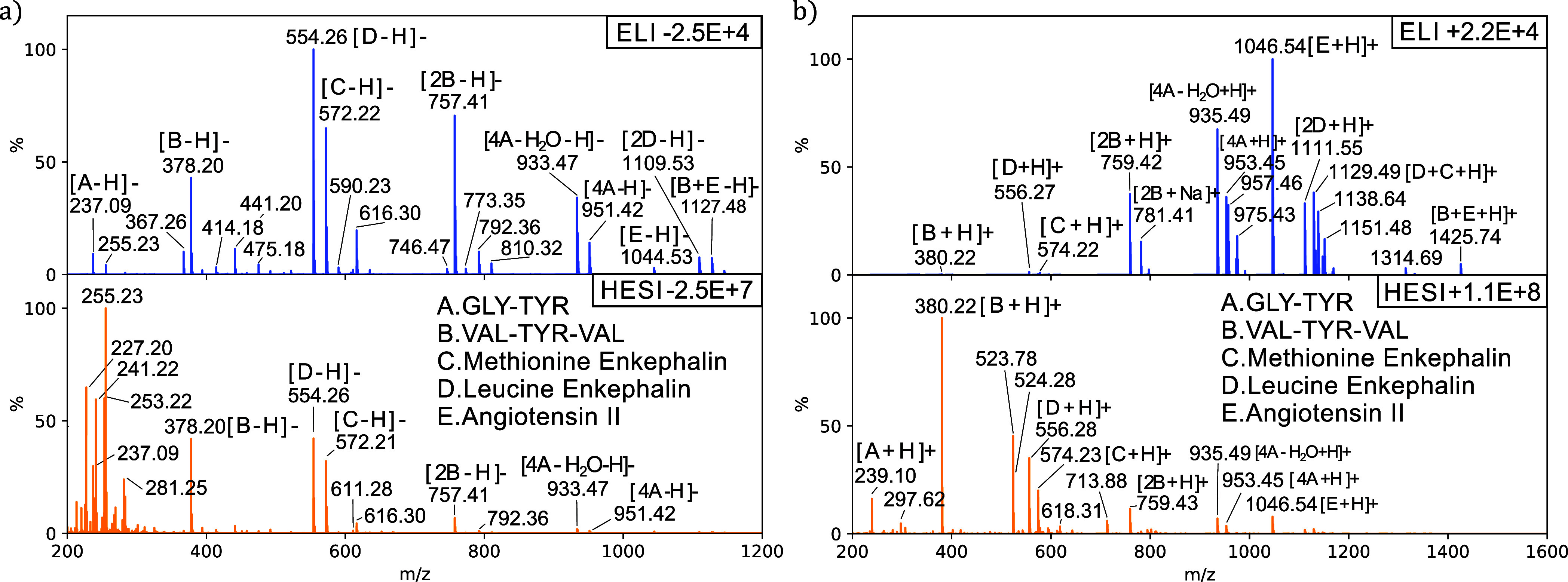
Mass spectra of a peptide mixture using HESI and ELI in negative (a) and positive (b) mode on the Q Exactive mass spectrometer (Orbitrap). The solution contains A/GLY-TYR (238.25 g mol^−1^), B/VAL-TYR-VAL (379.51 g mol^−1^), C/methionine enkephalin (573.68 g mol^−1^), D/leucine enkephalin (555.63 g mol^−1^), and E/angiotensin II (1046.19 g mol^−1^) in a 50/50 mixture of MeOH/H_2_O at a concentration of 5 μg mL^–1^ for each component.

The mass spectra for both ELI and HESI are depicted in Figure 4. In negative mode, ELI exhibits a significantly better signal-to-noise ratio than HESI, which could be due to fragmentation in the case of HESI, where numerous small mass-to-charge ratios are observed. In HESI, component E (angiothensin II) is not visible, while in ELI, all components as well as some clusters can be assigned. However, it should be stressed that the measurement was not optimized for HESI. For instance, increasing the pH to facilitate deprotonation could improve the HESI spectrum. In positive mode, the spectra are notably different as well, with relatively low-intensity molecular ion mass-to-charge ratios in the ELI spectrum and all molecular ions of the components clearly visible in the HESI spectrum. There appears to be a substantial presence of clusters and dimers for ELI in positive mode, of which some are tentatively assigned, e.g., dimers of component B ([2B + H]+, *m*/*z* = 759.42) and D ([2D + H]+, *m*/*z* = 1111.55) can be observed. As methionine enkephalin and leucine enkephalin are similar molecules, a cluster of both components, [C + D + H]+, would not be surprising, and, indeed, one can find *m*/*z* = 1129.49 in the spectrum, among many other clusters. The abundance of clusters for ELI in this mode could be attributed to the lower level of charging in comparison with HESI.

**Figure 5:**
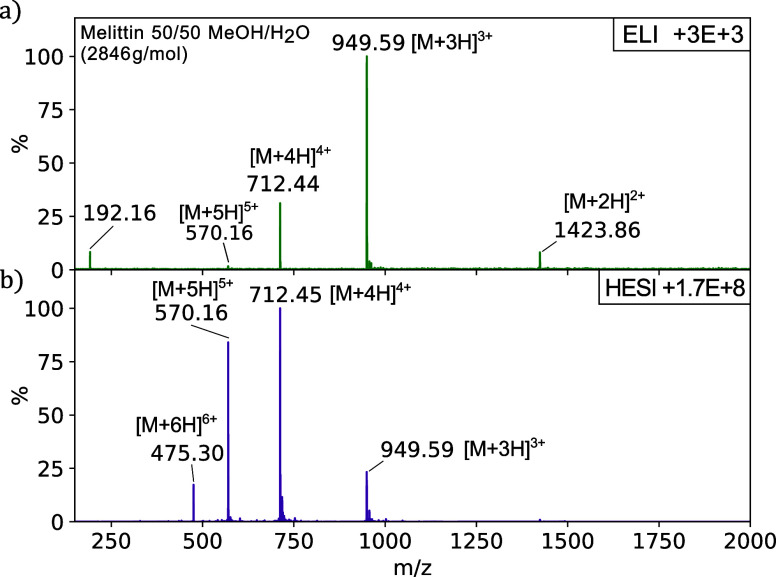
Mass spectra of bee venom (Melittin, 2846.46 g mol^−1^) in a 50/50 water–methanol mixture (16 μg mL^–1^), using ELI (a) and HESI (b) in positive mode as the ionization source for the Orbitrap mass spectrometer.

We also compared positive-mode ELI and HESI for single-peptide bee venom (Melittin, 2846.46 g mol^−1^), using 16 μg mL^–1^ within a 50/50 mixture of MeOH/H_2_O (Figure 5). The spectra are very similar, although the mass-to-charge ratios of multiply charged states differ significantly between ELI and HESI. Various factors may contribute to the observed difference, especially as ELI is an open-source technique while HESI is a closed source.

### Small Sample Consumption

**Figure 6:**
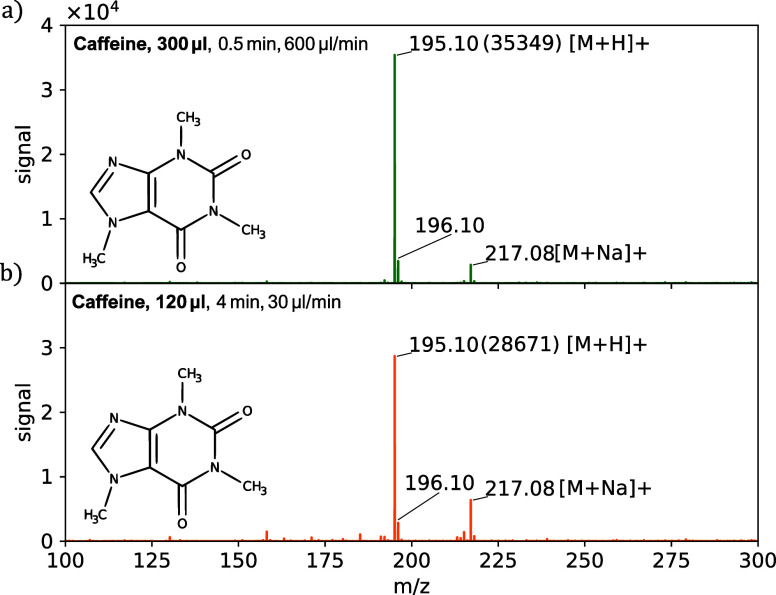
Mass spectra of caffeine (194.19 g mol^−1^) in 80/20 vol % water/ethanol (100 μg mL^–1^) at a flow rate of 600 μL min^–1^ for 0.5 min through a nozzle with 85 holes of 1.9 μm (a) and at a flow rate of 30 μL min^–1^ for 4 min through a nozzle with 8 holes of 1.9 μm. The molecular ion (*m*/*z* = 195.1) is the most abundant, and the sodium adduct can also be observed.

In many practical cases, the sample size is small, requiring a highly efficient ionization process and short-duration measurements. For LC–MS applications, a lower flow rate would be more desirable as well. In ELI, the nozzle design determines the flow rate. When using a nozzle with 85 holes of 1.9 μm, with a flow rate in the range 400–600 μL min^–1^, we find that a large part of the resulting spray plume does not enter the MS. Using a spray nozzle with less holes can make the ionization more efficient, though more effort is needed to correctly direct the smaller spray plume toward the MS inlet. To explore the effect of flow rate, we measured a solution of 100 μg mL^–1^ of caffeine (194.19 g mol^−1^) in water/ethanol (80/20) at 600 μL min^–1^ versus 30 μL min^–1^ flow rates. For the high flow rate, we use a nozzle with 85 holes of 1.9 μm (Figure 1d), while for the low flow rate, we use a nozzle with 8 holes of the same size. Figure 6 shows the mass spectra for a high flow rate (a) sampled for 0.5 min and a low flow rate (b) sampled over 4 min. Though the total sprayed volume for the low flow rate (b) is more than half of that for the high flow rate (a), the signal intensity is almost the same, demonstrating that the low flow rate resulted in a higher ionization efficiency. It should be noted, however, that the efficiency under ambient conditions could depend on many other factors such as the evaporation, positioning of the spray, external air flows, and electrical grounding.

### Quantitative ELI-MS

**Figure 7:**
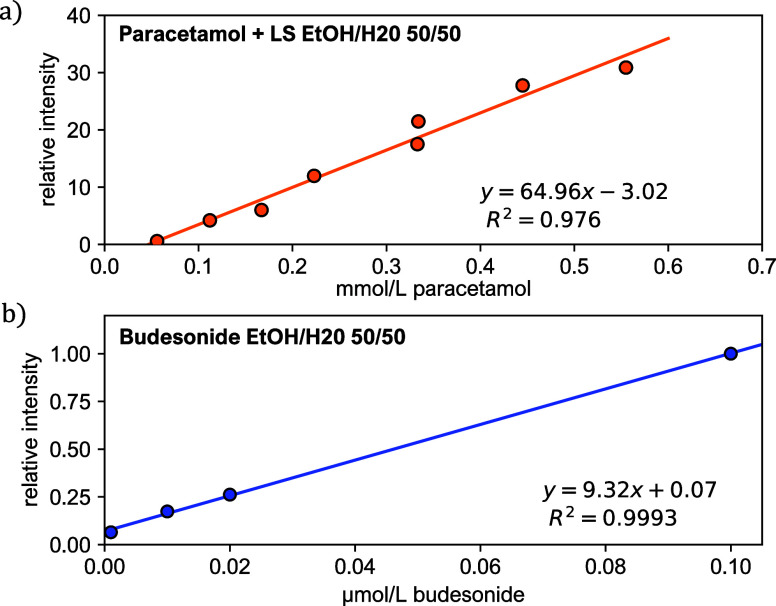
Linearity of ELI-MS. (a) Relative response for different paracetamol concentrations. The intensity of [M + Na]^+^ for paracetamol is rescaled with the peak of the sodium adduct of LockSpray (LS, leucine enkephalin; 555.62 g mol^−1^). The linear range for paracetamol in this case is 50–600 μmol L^–1^. (b) Linear response for budesonide, using the sodium adduct as the main signal. The linear range for budesonide is 10^–9^–10^–7^ M.

To obtain quantitative ELI-MS, the infused concentration and the ELI-MS response need to be correlated. This is extremely challenging because of well-known material related factors impacting quantitative MS (e.g., ionization efficiencies, analyte concentration, degradation, sample preparation, signal stability and repeatability, ionization interference, and detection spectrum^22,23^), as well as instrumental limitations (e.g., ion source parameters, ion transmission efficiency, detection efficiency, and instrument contamination). In the case of ELI-MS, we find spray interruptions caused by an accumulation on the nozzle of droplets that fly back due to electrostatic forces of the oppositely charged nozzle surface. In the absence of grounding, fluctuations in the total ion current are to be expected, besides fluctuations associated with the open ion source. In addition to spray irregularities for large flow rates (0.7 mL min^–1^), the charging efficiency can also be affected by the concentration of the compound and the presence of ions in the solution. In addition, the ELI-MS setup is installed in an open laboratory, where it is exposed to possible variations in the flow of air within the room. Therefore, we opt for an internal standard to observe the linear response of ELI-MS for quantitative applications. We used paracetamol (151.163 g mol^−1^) in combination with leucine enkephalin (LS: 555.62 g mol^−1^) as internal standard. Calibration lines were constructed by injection of different amounts of sample and the same amount of standard leucine enkephalin (1 ppm). Figure 7a shows the calibration curve of paracetamol calculated using standard addition. Linear relationships were found in the concentration range 50–600 μmol L^–1^ with correlation coefficient 0.984. Although this demonstrates the linearity of ELI-MS, the results differ somewhat from the literature reported values,^24,25^ which is likely due to matrix effects caused by using leucine enkephalin as an internal standard. Quantitative measurements could be improved by using an isotope-labeled internal standard. For budesonide (Figure 7b), which ionizes readily, a linear relationship could be found without the use of an internal standard. The linear range for budesonide in this case is 10^–9^–10^–7^ M, similar to literature reports.^26−28^ Additionally, we studied the matrix effect of the addition of leucine enkephalin to budesonide samples (see Supporting Information “Matrix Effects Leucine Enkephalin”).

### Desalination

Besides the properties of the nozzle, other parameters such as the conductivity of the liquid can affect the charging efficiency of the ELI method, as charge build-up cannot occur if the liquid is so conductive that separated charges simply flow back. To limit the conductivity of the fluids, salt concentrations should be low. Fortunately, desalination of samples is a common sample preparation step. By studying solutions of different concentrations of NaCl in water, we find that concentrations lower than 10^–4.5^ M do not affect the charging significantly. Similarly, the pH of the sample should not be lower than 4.5 or higher than 9.5. In LC–MS, the eluent typically lacks salts, which is beneficial for ELI, but makes ionization difficult with methods like ESI. This highlights the fundamental difference between ELI and direct charging methods like ESI.

### Test Case: Secondary Ionization of Perfume

**Figure 8:**
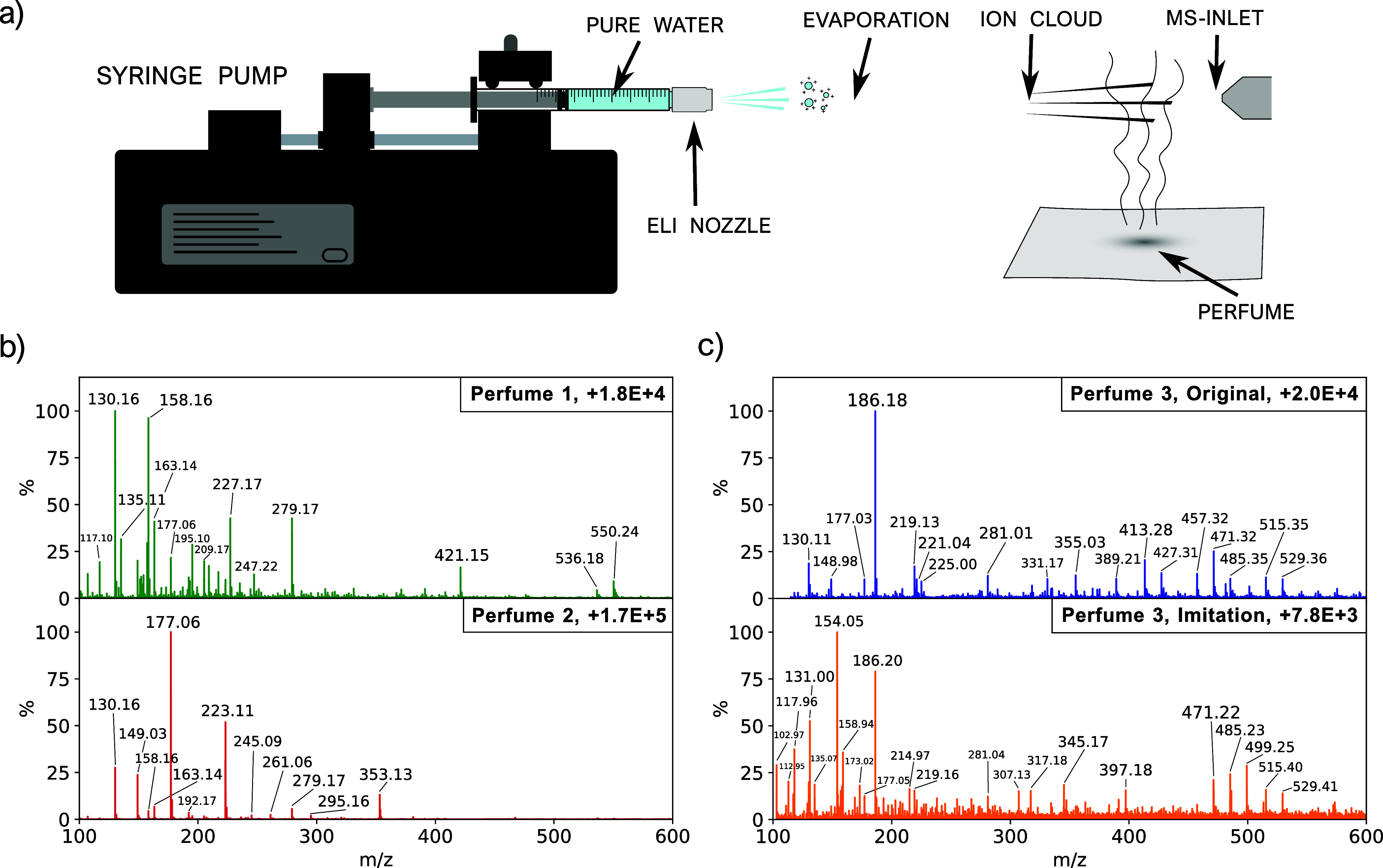
Secondary ionization of perfume samples using ELI. (a) Schematic setup of the experiment. (b) Mass spectra of two different perfumes (perfumes 1 and 2), measured using indirect ionization. The mass spectrum of perfume 2 shows the presence of DEP,^30^ with characteristic peaks at *m*/*z* = 149, 177, and 223. (c) Mass spectra of perfume 3 (top panel) and an imitation thereof (lower panel). While the two spectra clearly show some overlap, there are also notable differences, especially in the lower *m*/*z* range.

To demonstrate the potential of the ELI nozzle in other MS applications, we used it in an experiment similar to secondary electrospray ionization (SESI).^29^ Here, the ELI nozzle creates a cloud of ionized water droplets directed toward the MS inlet at a distance of approximately 20 cm (Figure 8a). This ion cloud subsequently ionizes the volatile components from perfume emanating from a piece of paper, allowing MS analysis. When comparing spectra across three conditions (spray only, spray with a piece of clean paper, and spray with a perfumed piece of paper), we only observe a clear spectrum in the third case. Figure 8b shows the mass spectra for two perfumes using ELI in positive mode, highlighting the potential of the ELI-SESI method to fingerprint perfumes. The spectrum of perfume 2 seems to be completely dominated by diethyl phthalate (DEP),^30^ with characteristic peaks at *m*/*z* = 149, 177, and 223, even though this controversial ingredient is not included in the list of ingredients printed on the bottle. To further highlight the method’s potential, Figure 8c presents the spectra of a third perfume and imitation thereof. While there is evident overlap between the two spectra, discernible differences emerge, particularly in the lower *m*/*z* range.

### Test Case: Fast Fungicide/Pesticide Screening of Citrus Fruits

**Figure 9:**
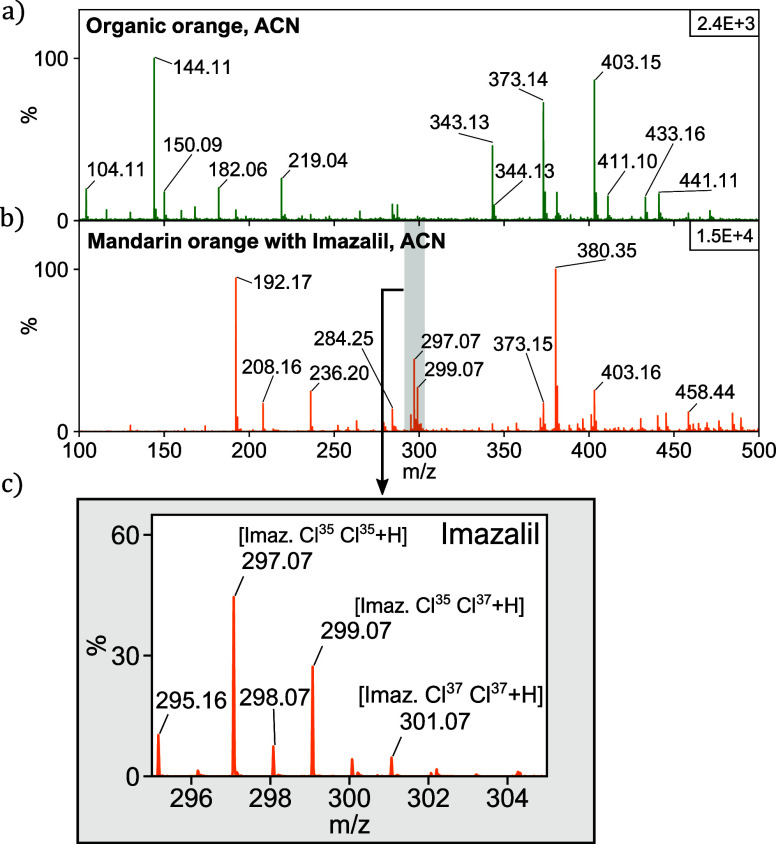
Mass spectra of the peel of an organic, untreated orange (a) and a store-bought, imazalil-treated orange (b). (c) Zoom-in of the characteristic peaks of imazalil for the treated orange: RCl^35^Cl^35^, RCl^35^Cl^37^, and RCl^37^Cl^37^ with 57.4, 36.7, and 5.87% abundance, respectively.

Finally, we highlight the potential of ELI for fast MS screening of citrus fruits. These are often waxed and treated with fungicides such as imazalil to prevent the formation of mold. While the use of imazalil is regulated, the substance is labeled as ‘suspected of causing cancer’.^31^ We studied mandarin oranges purchased at a local supermarket, which according to the label have been waxed and treated with imazalil, as well as organic oranges taken directly from a home-grown orange tree. The spectrum of the store-bought orange clearly shows the characteristic peaks of imazalil, where the relative abundances correspond with the signal intensities for each peak. These peaks are absent in the spectrum for the organic, untreated orange (Figure 9). The remaining peaks in the spectrum of the organic orange do not seem to correspond with the ones in the treated oranges. One of the possible reasons for this is that the frozen samples probably released more intracellular compounds. Also, the treated orange contains an artificial wax layer, which is likely to be responsible for other peaks in the spectrum.

## Conclusions

We introduced a new technique to generate gaseous ions for MS that requires no electronics to function. The so-called ELI technique makes use of the electrokinetic interaction of the fluid with the walls of the microfabricated nozzle through which it is pushed, allowing to charge almost all commonly used solvents for MS analysis, such as water, water/ethanol, water/methanol, acetonitrile, and dimethyl sulfoxide. The polarity of the ionization can be changed by adapting the charge transfer layer, in this case, by employing a different nozzle. One of the notable advantages of ELI-MS is that the ionization source is very compact, which could prove useful in the integration of this technique in other applications, such as LC–MS. Adjusting flow rates is also easy for ELI-MS by changing the liquid pressure or number of nozzle holes. Additionally, the use of disposable syringes and mass-fabricated ELI nozzles eliminates the need for time-consuming cleaning procedures. These characteristics and the ease of use make ELI-MS especially advantageous for on-site/fieldable MS applications.

To demonstrate the new technique, we used it as a stand-alone ionization source but also as part of other MS techniques such as secondary ionization. We analyzed a wide variety of components using various solvent mixtures, ionization polarities, and mass spectrometers. More research is needed to explore all the different aspects of ELI-MS. Some promising directions are the combination of ELI with LC–MS, the use of different charge transfer layers, compound-releasing charge transfer layers, the ionization of fragile molecules, and optimizing the ionization efficiency by adjusting flow rate, nozzle chip, and electrical grounding.

## Supporting Information Available

Measured ion currents for grounded and nongrounded ELI nozzles and matrix effects with ELI-MS (PDF)
