# Supplementary material for: Electroless Ionization Mass Spectrometry Using a Compact Electrokinetic Ionization Source
Source: Anal Chem. 2024 Jun 25;96(27):10978–85. doi: 10.1021/acs.analchem.4c01403 (PMC11238159; doi:10.1021/acs.analchem.4c01403)
Supplement: Supplementary file 1 — ac4c01403_si_001.pdf [file ac4c01403_si_001.pdf]

Supporting Information

Charging stability and grounding

In all the experiments the ELI source has been used as an ambient technique. Variations in the ion current, due to factors such as air currents, are to be expected. To keep the setup as simple as possible, the ELI nozzles used in the experiments are not grounded. We find that the setup as a whole provides enough electrical conduction to maintain a sufficient level of ionization for the period of the measurement. However, this could depend on the materials used and on the environmental conditions such as humidity. Additionally, droplet accumulation on the nozzle driven by electrostatic forces, can cause spray interruptions. To explore this dependency without introducing other variations associated with ambient MS, we measured the ion current externally using an electrometer (Keithley 617). We used a 1 mL plastic syringe, delivering a flow rate of 0.7 mL/min via a syringe pump. The spray is directed to the inside of a Faraday cup, that is connected to the electrometer. For the non grounded nozzles we use the regular ELI nozzle which consist of a polypropylene adapter. For the grounded nozzle we use an ELI nozzle adapter, which has been sputter-coated with gold on both the inside and outside of the adapter. The nozzle chip is however free of any gold layer. Figure 1 displays the ion current produced by both configurations.

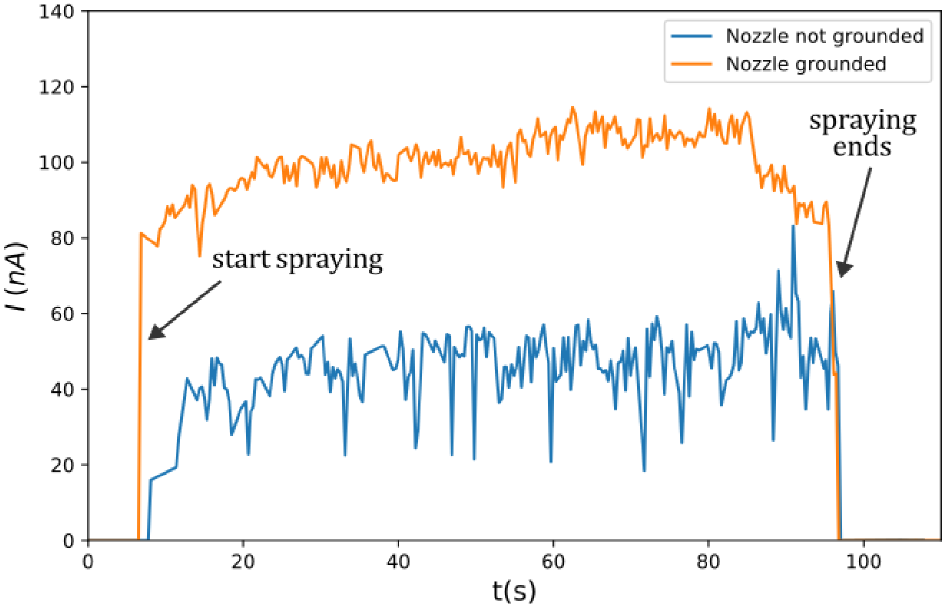

Figure 1 Measured ion currents for a grounded and non grounded ELI nozzle. The start and end indicate the beginning and end of the spraying process.

The data clearly show that the grounded nozzle achieved a higher and more stable ion current. In contrast, the non grounded nozzle exhibited pronounced spikes, likely due to droplet accumulation on the adapter.

Matrix effects leucine enkephalin

We investigated the matrix effect of adding leucine enkephalin as an internal standard to budesonide samples. Figure 2 illustrates how the inclusion of leucine enkephalin impacts the budesonide response. We found that the sensitivity was affected at higher concentrations of internal standard present in the sample. At high standard addition, the molecular ion almost disappears and the signal of the leucine enkephalin is most abundant. However, at 10 times lower concentration of leucine

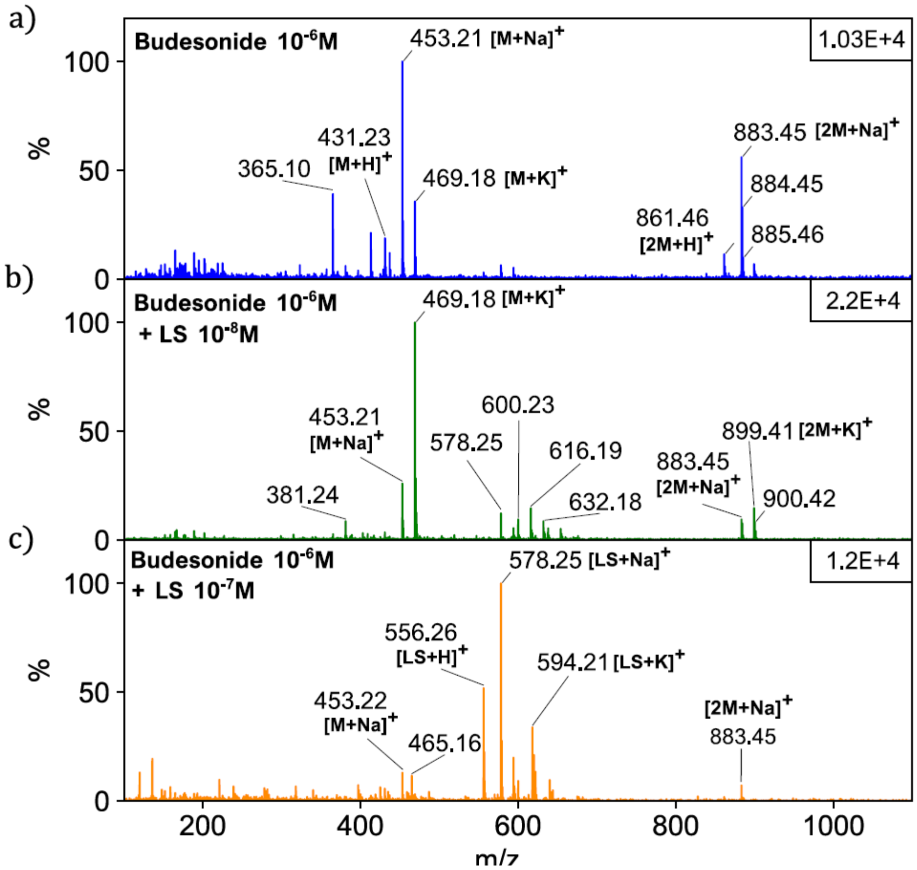

Figure 2 Matrix effects with ELI-MS. a) Budesonide 10^{-6} M. The most abundant peaks are the sodium adduct $[M + Na]^+$ and the cluster $[2M + Na]^+$. b) Addition of 10^{-8} M leucine enkephalin. The sodium adduct $[LS + Na]^+$ can be observed at m/z = 578.25. c) Addition of 10^{-7} M leucine enkephalin. The budesonide signal is suppressed and the most abundant peak is of leucine enkephalin, $[LS + Na]^+$.

enkephalin (Figure 2 c), the effect on the budesonide signal is no longer significant and the sensitivity is not affected by presence of the internal standard. This demonstrates the ion suppression/competition phenomena and their major influence on the quality of the spectra. Though the ionization mechanism in ELI is distinctly different from that of ESI, once charged droplets with analytes are formed, the same physics applies, and one can expect the same difficulties with complex mixtures for ELI-MS. The combination of an ELI nozzle with a separation column could therefore be an attractive one, especially as it offers several advantages over regular LC-MS, such as the small dimensions of the ELI-chip and the ability to have good ionization for low conductive liquids.
